# Next Steps for Ebola Vaccination: Deployment in Non-Epidemic, High-Risk Settings

**DOI:** 10.1371/journal.pntd.0004802

**Published:** 2016-08-18

**Authors:** Laura A. Skrip, Alison P. Galvani

**Affiliations:** Center for Infectious Disease Modeling and Analysis, Yale School of Public Health, New Haven, Connecticut, United States of America; Walter and Eliza Hall Institute, AUSTRALIA

The catastrophic West Africa Ebola epidemic that resulted in approximately 28,200 cases and 11,300 deaths over 22 months has been reduced to intermittent and, thus far, rapidly controlled flare ups in Liberia, Guinea, and Sierra Leone. During the period of most widespread and intense transmission, extraordinary efforts to develop, produce, and field-test an Ebola vaccine in the outbreak setting generated promising results. The combined findings of four parallel Phase I studies on healthy adults in Gabon, Kenya, Germany, and Geneva demonstrated safety and immunogenicity of the recombinant vesicular stomatitis virus (rVSV)-vectored *Zaire ebolavirus* (rVSV-ZEBOV) vaccine [1]. Glycoprotein-binding antibodies were detected for all vaccinated participants irrespective of dose and, although arthralgia, fever, and maculopapular rash were correlated with immunization, the mild symptoms were transient and easily treated; the vaccine was deemed safe with a high likelihood of acceptance [1]. More recently, it has been suggested that the rVSV vaccine effectively protects against Ebola virus disease (EVD) infections among the contacts of symptomatic cases in intense transmission settings [2,3]. Other candidates include the chimpanzee adenovirus type 3 Ebola vaccine (cAd3-EBO), which has been demonstrated as immunogenic and safe in humans [4]. Additionally, sustained protection against lethal Zaire ebolavirus (EBOV) challenge in macaques was achieved using ChAd3, given a modified vaccinia Ankara (MVA) boost after two months [5]. Largely mediated by CD8+ cells, a protective effect against EBOV has likewise been established in macaques for a vaccine based on recombinant adenovirus virus serotype 5 (rAd5) encoding Ebola virus glycoprotein (GP) [6–8]. Across all vaccine candidates [9], large-scale trials in human populations are unlikely to yield sufficient statistical power for determining efficacy and duration of protective immunity given the fortunate downturn in transmission events. However, if an Ebola vaccine is approved for post-marketing surveillance without additional Phase III efficacy studies, there is an opportunity to prepare vaccine infrastructure, including stockpiling and administration systems, in non-emergency but high-risk settings.

Ebola emergence in human populations has become a sporadic yet recurring event. Thirteen African countries constituting over one-fifth of the continent have been affected by at least one documented outbreak since 1976 [10]. Although use of reactive vaccination in outbreak settings has been demonstrated to be effective in conjunction with extensive surveillance and contact tracing that took many months to implement during the recent outbreak [3], proactive vaccination could have the potential to mitigate future zoonoses if appropriately targeted.

Phased vaccination of health care workers in highest risk countries could protect otherwise fragile health systems in the event of Ebola emergence. Approximately 880 health care workers were infected in the West Africa outbreak and nearly 60% died [11]. In Liberia, Sierra Leone, and Guinea, where there is only one physician for more than 20,000 people on average, routine vaccination against Ebola could provide critical protection to safeguard an already inadequate number of medical workers [12]. In addition to the direct protection for medical staff, sufficient coverage in hospital settings would reduce nosocomial transmission (e.g., [13]). Throughout the epidemic in West Africa, occupational risk associated with health care settings, even those not designated for Ebola treatment, led to decisions to miss work. It has been estimated that externalities of the inaccessibility of health care generally during the outbreak may have been even greater than the direct impact due to Ebola-specific mortality [14].

**Fig 1:**
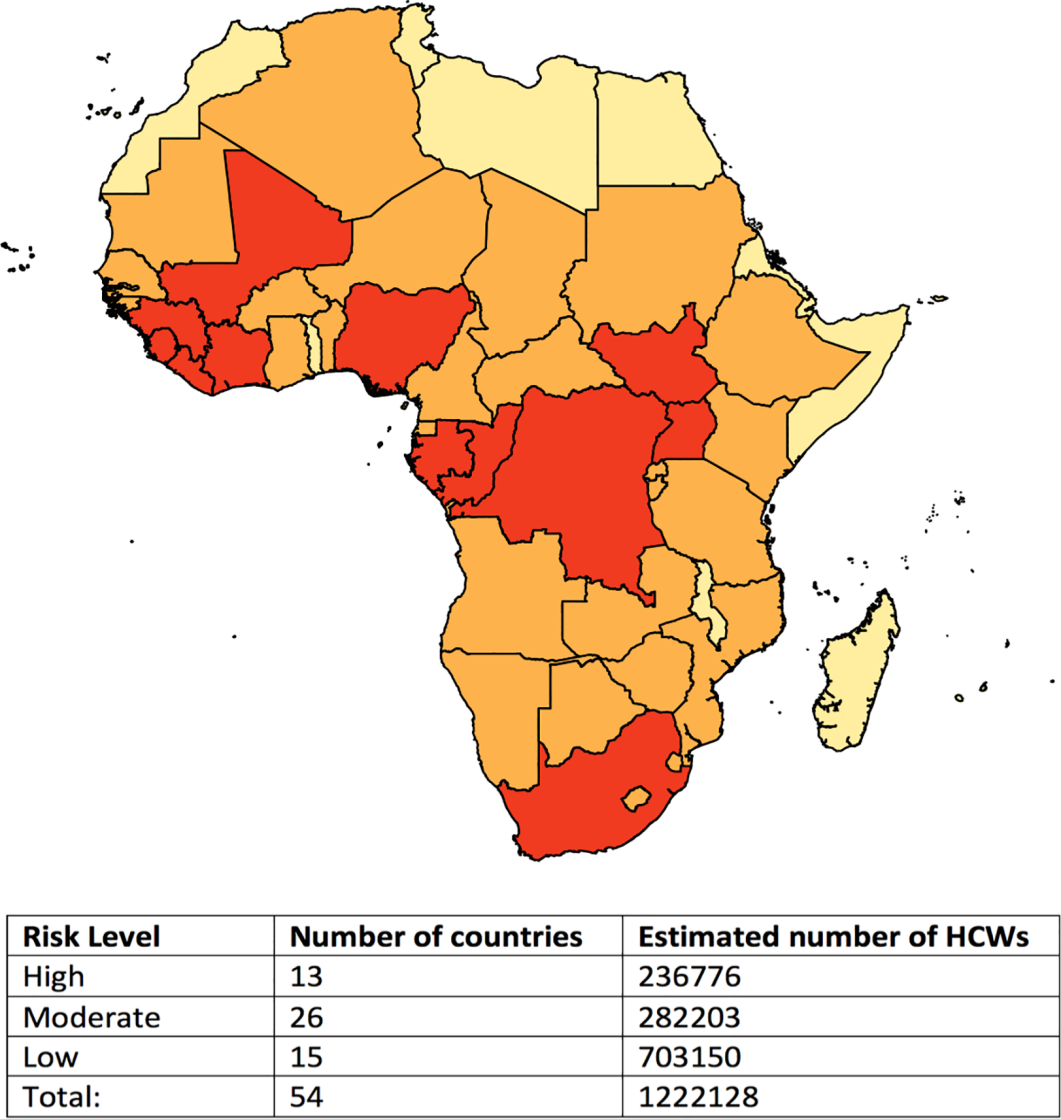
Ebola risk level of African countries based on history of outbreaks. Countries at highest risk (red) of a future Ebola outbreak were identified as those affected by at least one documented outbreak since 1976. Moderate-risk countries (orange) are those with borders contiguous to high risk countries, and low-risk countries (yellow) are those with no history of Ebola cases and without shared borders with high risk countries. Health care worker (HCW) estimates were derived from the most recent available World Bank estimates of numbers of physicians, nurses, and midwives (per 1,000 population) and the 2014 population in each country.

The term health worker is typically used to refer to doctors, nurses, and midwives [15–17]. Based on country-specific estimates from World Bank and World Health Organization (WHO), there are approximately 1.2 million physicians, nurses, and midwives in the 54 countries on the African continent [18,19]. Just over 236,000 of these health care workers are located in the 13 countries that have experienced at least one documented Ebola case and are likely at highest risk of Ebola zoonosis (Fig 1) [10]. In addition, 26 countries share a contiguous border with a high-risk country and are thus considered at moderate risk. With current stockpiles of over 150,000 doses of the rVSV-ZEBOV vaccine as well as recent production of 9,000 for the trial in Guinea and 6,000 for the STRIVE trial in Sierra Leone, sustaining current production capacity would be sufficient for phasing in the proactive approach of vaccinating health care workers in high-risk settings while building a stockpile for reactive deployment in the event of an outbreak. For instance, production capacity of 9,850 doses per month would be enough to vaccinate health care workers in all high-risk areas within 24 months and all moderate-risk areas over a subsequent 30 months. The availability of an Ebola vaccine stockpile has recently been ensured through funds designated by GAVI, the Vaccine Alliance, to Merck for production and maintenance of 300,000 rVSV-ZEBOV doses and regulatory approval [20].

Administration across high-risk countries could be conducted according to a prioritization scheme determined by a combination of indicators. Such indicators would account for recency of the last known transmission event as well as capacity for maintaining and distributing the vaccine supply, monitoring of safety concerns, and evaluating effectiveness or duration of protective immunity. It is recognized that involvement of an international authority, such as WHO, would be required to obtain and allocate the vaccine among countries, while individual countries would establish regional policies for implementation of a prescribed vaccination strategy. National policy would accordingly specify efforts to maximize participation and ensure adequate monitoring and evaluation based on international protocols.

Phased implementation at the country level could involve randomly predetermined clusters of health facilities vaccinated sequentially as part of a stepped wedge design [21]. Facilities in an administrative health district could form a single cluster, for instance. Monitoring of vaccinated health care workers would therefore provide larger scale data on safety and immunogenicity. For the former, regular assessments of adverse effects could be conducted by a designated provider at each health facility with vaccinated employees and submitted to the appropriate authority. The frequency of assessments would initially follow standard Phase I procedures, but less often assessments could continue beyond the first months of follow-up. Furthermore, the use of an appropriate phasing design would not simultaneously jeopardize the entire workforce if previously unobserved side effects were to develop. In terms of immunogenicity, twice annual blood draws could provide information on antibody response, although appropriate measurement of cellular immunity may be required to assess longer term protection [22]. Ongoing discussions will be necessary to identify methods of monitoring cellular immunity, and samples acquired during repeated draws from vaccinated individuals should be maintained for future testing, if appropriate.

The approach of vaccinating health workers is potentially more feasible than other mass vaccination campaigns because it targets a relatively well-defined and receptive population. Although suspended in the absence of active transmission, vaccination of 6,000 health care and other frontline workers in seven urban and rural areas of Sierra Leone had been undertaken as part of the STRIVE trial. The recruitment strategies and enrollment milestones designed for this study could inform the development of routine vaccination protocols. A convenient administration strategy could be the vaccination of nurses and physicians during their training. Because duration of health care worker occupation is typically several decades, boosters may be necessary to sustain protection in the event of waning efficacy. In countries with little turnover within the profession, high coverage rates would be maintained without requiring large-scale “catch up” campaigns. Furthermore, the health care workforce is expected to have higher vaccine acceptance. During the Ebola June 2015 resurgence in Monrovia, physicians and health workers from hospitals where cases were being treated and/or contacts were being quarantined have opted to receive the vaccine [23].

Despite the advantages of using health care workers as a targeted population for mass administration of the Ebola vaccine, inter-country variation in the stability of the population presents some potential drawbacks. The persistent shortage of health workers in many sub-Saharan African countries has been attributed to insufficient national investment in training systems, as well as out-migration due to wages, transition to different professions, and fear of occupational transmission, particularly of blood-borne pathogens such as HIV and hepatitis B [24]. In countries with significant attrition among health workers, a brief assessment could be taken to determine the point in providers’ careers at which out-migration is most probable and prioritize vaccination for individuals for whom that is least likely, for example, as might be correlated with number of years in clinical practice. Alternatively, if vaccination were provided at the time of pre-service training, individuals could be asked to opt out if they did not intend to participate in residency or start a nursing or midwifery position postgraduation. Due to the general shortage of health workers and the accompanying high rates of disease, countries with the greatest risk of Ebola tend to experience a regular influx of international aid, including human capacity. It would be expected that clinical visitors be vaccinated according to the policies of their host countries and within the protocols of the overarching vaccination program. For instance, health workers who move into districts in which such as vaccination program was already implemented should likewise be offered the vaccine.

Other mass vaccination strategies intended to reduce secondary transmission events may be complementary. Through an initiative headed by WHO, Guinea is currently preparing to vaccinate contacts of survivors in an effort to safeguard against the flare ups that have been rattling West Africa since July 2015 [25]. Similar to vaccinating health workers, this approach is targeting not only a high-risk group but also an evolving population, as survivors’ contact networks are inevitably dynamic. Our understanding of the duration of viral persistence in survivors as well as the durability of protective immunity afforded to humans by the vaccine is still developing, such that the anticipated effectiveness of the approach has several uncertainties. In addition, allocation of the vaccine stockpile to potential super-spreaders, such as community leaders or members of large households, could be an effective approach specifically in regions with ongoing or very recent transmission. However, a challenge is the accurate identification of individuals likely to be super-spreaders. These approaches would likewise benefit from a phased administration strategy, although the feasibility of a complicated study design may be more challenging in community-based settings than health care settings.

Due to the combination of behavioral change, active epidemiological surveillance [26], expanded hospital bed capacity [27], and other response efforts, a rapid decline in incidence restricted the number of high-transmission settings suitable for conducting vaccine trials during the 2014–2015 Ebola epidemic. Criticism of the analysis applied for the ring vaccination trial in Guinea, including imbalances in cluster sizes between trial arms and *p*-values that did not account for α-spending rules, has advised caution in interpreting the reported efficacy [28]. Prior to widespread use of the vaccine, further Phase II and III trials have been advocated [14]. However, an epidemic of similar magnitude that could yield meaningful statistical evidence will hopefully be averted by lessons learned as well as the Ebola vaccines that were not available when the recent outbreak took hold. Additionally, pooling evidence from a series of smaller clinical trials raises challenges, such as between-study heterogeneity in sample size, risk of exposure, and magnitude of effect [29].

Extraordinary scientific and humanitarian momentum was garnered during the 2014–2015 Ebola outbreak. Routine vaccination of health care workers in non-epidemic but high-risk settings could be an effective approach for sustaining this momentum. We recognize that the approach includes challenges, such as possible inter-epidemic waning of protection, a large population of health care workers who are not nurses, midwives, or physicians, and poor coverage among health care workers in more rural areas or regions less accessible due to political unrest. In addition, criticism of the ring vaccination trial conducted in Guinea has prompted uncertainty regarding the accuracy of the estimated efficacy [28]. Nonetheless, examining proactive vaccination strategies for Ebola prevention and response is critical to advancing development of vaccine production and administration technologies that could be instrumental to mitigating future Ebola outbreaks.
